# Guidance for Acupuncture Robot with Potentially Utilizing Medical Robotic Technologies

**DOI:** 10.1155/2021/8883598

**Published:** 2021-03-31

**Authors:** Tiancheng Xu, Youbing Xia

**Affiliations:** ^1^Key Laboratory of Acupuncture and Medicine Research of Ministry of Education, Nanjing University of Chinese Medicine, Nanjing, China; ^2^Affiliated Hospital of Xuzhou Medical University, Xuzhou Medical University, Xuzhou, China

## Abstract

Acupuncture is gaining increasing attention and recognition all over the world. However, a lot of physical labor is paid by acupuncturists. It is natural to resort to a robot which can improve the accuracy as well as the efficacy of therapy. Several teams have separately developed real acupuncture robots or related technologies and even went to the stage of clinical trial and then achieved success commercially. A completed clinical practical acupuncture robot is not far from reach with the combination of existing mature medical robotic technologies. A hand-eye-brain coordination framework is proposed in this review to integrate the potential utilizing technologies including force feedback, binocular vision, and automatic prescription. We should take acupuncture prescription with artificial intelligence and future development trends into account and make a feasible choice in development of modern acupuncture.

## 1. Introduction

Acupuncture is gaining increasing attention with the emergence of integrative medicine. Combination of modern technologies and traditional acupuncture will bridge the cultural gap with Western medicine and improve the development of integrative medicine. The current advancement of the acupuncture robot and the potential utilizing medical robotic technologies are summarized in this review.

Medical robotics has rapidly become a rich and diverse area of research, and acupuncture robot is not an exception. Nowadays, many different institutions have individually developed their own form of acupuncture robot and numerous modern technologies have been developed for acupuncture robot, such as binocular vision for acupoint location and force feedback for needle insertion.

**Figure 1:**
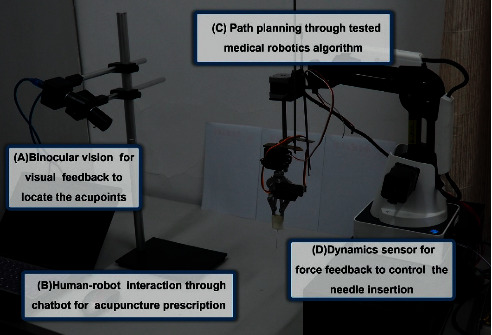
Prototype of medical acupuncture robot.

It is a comprehensive work to develop an acupuncture robot of clinical practical value. Firstly, the automatic location of hundreds of acupoints demands the integrated work between robotic arm and cameras, shown as part A in Figure 1. Secondly, the insertion of needle should be fast as well as stable to make the treatment effective. Thirdly, the evaluation of Deqi which is a special feeling that ensures the curative effect of acupuncture must be conducted through the robot system, shown as part D in Figure 1. Last but not least, the acupuncture robot should prescribe for patients automatically, shown as parts B and C in Figure 1. In summary, the final version of a practical acupuncture robot may meet those criteria and under the administration of the government. The criteria vary among countries which demand more effort of the inventors.

It is natural to resort to robots to perform acupuncture work for their precision and endurance. According to the published research, the earliest idea about acupuncture robot was mentioned as high-tech acupuncture by Gerhard Litscher of Medical University of Graz in1997 [1]. More than twenty years ago, when the research team at the Medical University of Graz, in 1997, showed that acupuncture also works in the electronics laboratory without the aid of an acupuncturist and when they introduced the term computer-controlled acupuncture [2], they did not use the term meaning that the computer replaces the acupuncturist; they rather understood the quantification of measurable effects of acupuncture [3]. Meanwhile, the vision with robotically assisted acupuncture seems to have become a reality [4, 5]. However, there has been no complete theoretical guidance for the development of such a robot so far, and many technologies for acupuncture robot are attributed to surgical robot. Those works include needle steering for lung biopsy and prostate biopsy, which can be feasible for acupuncture robot, so we include them in this review.

Figure 1 shows the prototype of a medical acupuncture robot developed by Dongdong Lu, Tiancheng Xu, and Boyu Jia with patent number ZL201610638071.3 [6], including parts A and C to locate the acupoints and move the robot arm which can insert the needle automatically and parts B and D to enable multilevel communication to facilitate information exchange between robot and users with sensors and chatbot.

## 2. Potentially Utilizing Medical Robotic Technologies of Acupuncture Robot

The process of acupuncture treatment can be divided into seven stages: diagnosis and prescription, acupoint positioning, topical disinfection, needle insertion, manipulation, and needle withdrawing, sometimes it is needed to stop bleeding by applying pressure to the acupoint. As we have discussed above, some features are commercially available, and in this review, we mainly focus on the following components: (a) automatic acupoint localization; (b) needle insertion; (c) acupoint stimulation; (d) automated detection for the efficiency of acupoint stimulation; (e) acupuncture prescription with artificial intelligence.

### 2.1. Automatic Acupoint Locating

Precise targeting is essential for adequate treatment of stimulation during acupuncture therapy, which requires a relatively a high level of skills and experience of the practitioners. Accuracy of point location is essential for safe, efficacious, and reliable treatments and valid reproducible research outcomes [7, 8]. These features are unique to acupuncture practitioners. Almost all hospitals are treating patients with artificial acupuncture by experienced traditional acupuncturists, who pay a lot of physical labor. In other words, there is still a shortage of instruments that can detect and locate acupoint. Lack of training and increased workload are great barriers for junior acupuncturists [9]. It is particularly dangerous for acupuncture therapy because inaccurate location of acupoints can cause even death like a needle penetrating the pericardium [8]. Many of the serious adverse events of acupuncture might have been caused by substandard practice [10]. It is of great practical value to develop automatic acupoint-locating devices which can help or even replace manual location in clinical application.

#### 2.1.1. Phantom Models and Animal Experiments

Generally, acupoints are classified into one of the three types according to the method used to locate them: anatomical points, proportional points, and morphological points. Anatomical points can be found with the help of bones and muscles or their specific crevices. Proportional points were calculated from the positions of anatomical points. The morphological points are also calculated by using some control points related to the connections between the source and the target models. Traditional acupuncturists invented the bronze man for the location of numerous acupoints, which is still in use today [11, 12].

Nowadays, new bronze man is being developed in the form of a phantom model [13]. Phantom models are commonly used to test the performance of the emerging image-guided targeting technologies. Variability in acupoint location will hamper the ability of researchers and clinicians to make meaningful comparisons among patients, while anatomic criteria for points based on objectively verifiable structures will facilitate translational research [14]. Fiducials are placed in anatomic gelatin phantoms and targeted by robotic systems [15], which can be feasible for the accurate marking of anatomical points. For the proportional points and morphological points, an acupuncture device can adapt its frame to new positions that are at relative distances from the position references in proportion with the extent of adjustment of the frame [16]. Phantom experiments indicate that robotic manipulation is equivalent in accuracy to manual insertion [17, 18]. More experiments on animals have been implemented to identify the acupoints on *Boa constrictor* and other species [19]. Even though the traditional Chinese and transpositional methods of animal acupoint location result in different acupoint charts, practitioners of both methods appear to achieve equally effective therapeutic results [20]. On this basis, more experiments are needed for humans.

#### 2.1.2. Human Experiments

Several designs [21, 22] of the automatic acupoint location system for humans have been developed. Those systems often consist of modules such as machine vision [23], augmented reality [24], and parallel positioning mechanism [25] to localize the acupoints. Researchers make use of a standard image inclusive of a 3D image for an internal tissue of the human body to produce a standard template for determining a standard location of acupoints [26]. A 3D virtual human body shape which is visible, regulated, and has human acupoints can guide the robot to carry out scientific and effective treatment according to the point information [27]. To locate the standardized 361 acupoints on the human body, digital data from a healthy Korean male were obtained by computed tomography [28]. A more systematic positioning method to precisely position acupoints including a computational algorithm for partial automation of the positioning [29] can make up for the weakness for the system.

More sophisticated technologies including reinforcement learning for image processing have been applied for the computer-aided automatic acupoint alignment [30]. Q-learning is adopted to optimize the robot positioning with little real-time computation [31]. Images of the thumb and middle finger of the body and local images of the limbs are collected by using the camera, which enables the machine to calculate the 3D coordinates of acupoints [32]. Such a system with high accuracy through integrating multiple techniques including landmark detection, image deformation, and the 3D morphable model was developed and tested. The mean error of estimation using the 3D fitted model is 8.16 pixels [33]. The method of positioning using photogrammetry methods based on the analysis of specially created unique square-shaped markers can calculate the spatial and angular positions of the robot [34]. Based on binocular stereo vision, the robot positioning acupuncture experiment shows that the accuracy of the method is within the allowable range [35].

More devices are integrated with automatic acupoint positioning with fruitful results [36]. Acu Glass based on Google Glass can draw the acupoints on top of the input face based on the height and the width of the user's face and the distance between the eyes [37]. However, such system cannot adapt to different face shapes, and their results are sensitive to the angle of the frontal face for robot-assisted acupuncture [38]. The infrared thermal image of the face is analyzed to mine the generalization feature of statistical distribution and then combined with automeasurement of the body to find the location of facial acupoint. Experiments on animals and human prove that the method is scalable for different human groups [39].

Although more study is needed, these results suggest that acupoint positioning is one of the several potential variables that should be considered in the optimization of user experience when performing automatic needling. Automatic acupoint locating may modernize acupuncture research and enable acupuncture treatments to be more personalized.

### 2.2. Path Planning, Needle Insertion, and User Interaction

Features such as acupoint positioning need to be integrated with a flexible and high-efficient systematic frame [40]. A medical robotic system usually comprises a robotic arm, a control system, and a secondary control module [41, 42]. To maximize the probability that the needle will reach the desired acupoint to ensure safety, stability, and robustness, the acupuncture robotic system should be equipped with various control features. Varieties of encoders and force sensors are needed to be installed to avoid substantial risk by a single fault. Parallel kinematic machines allow for better dynamic performance than serial machines [43]. The decomposition of differential kinematics into robot-specific and robot-independent portions is of great significance [44]. We address formulations at the velocity, acceleration, and force levels [45].

#### 2.2.1. Path Planning

The path trajectory of acupuncture needle insertion should be based on the best practices that are used by doctors [46] and tested through experiments. These planning algorithms can be integrated with emerging needle imaging technology to achieve closed-loop guidance and control of steerable needles [47]. As mentioned before, the phantom model is helpful to test the performance of emerging image-guided targeting technologies [15]. In terms of path planning, a location algorithm is based on simplified acupoints, where the self-organizing feature map neural networks and pattern recognition are combined to simplify the treatment spots to several classes. A series of discontinuous points are chained to a motion path [48]. However, tasks are complex and unique—each patient is different—and acupuncture treatment takes place in a nonstructured environment. Workspace is limited and must be shared with the patient and acupuncturist.

Robotics has been used for accurate surgical planning in various types of surgery [49]. Acubot (FDA clearance IDE G010331/S1), a highly dexterous, 7 degree-of-freedom robotic arm for CT-guided percutaneous needle biopsy, allows for an expansive working area and numerous approach angles to target all. The serial link design contributes to the robot's high dexterity while minimizing the profile [50]. The mean distance from the desired target for AcuBot was 1.2 mm [51], which is considered as a success reference for an acupuncture robot.

#### 2.2.2. Acupuncture Needle Insertion

Acupuncture has contributed to helping clinicians solve problems that are not being achieved in everyday life through the path of common medicine. What makes acupuncture unique is that the insertion of needles. Such technology tends to be mature in the field of medical robot, such as the robot for radiological percutaneous interventions with CT for needle biopsy [51]. Needle steering is a commonly used technique in the medical field as it enables doctors to reach the target tissue more precisely [52]. There has been much interest within the biomedical engineering community in needle steering to improve its accuracy.

As mentioned above, design of an acupuncture robot may include several technologies from other kinds of medical robots, especially for needle insertion control. Those medical robots can insert needles deeply into different kinds of tissues with higher standard than that of an acupuncture robot. To achieve needle steering robotically, the classical control strategies from those robots have to be rethought for acupuncture needle insertion. Accurate path planning and control of needle steering require models of needle-tissue interaction [53, 54]. For robotically steered needles, an analytical model that predicts the interaction forces and deflection of the needle is desirable for optimization of system design and real-time control [55]. Given a medical image with segmented obstacles and target, the robot can formulate the planning problem as a Markov decision process based on an efficient discretization of the state space [56]. Experiments showed an estimation of possible needle deflection based on manipulating the biopsy needle [52]. An insertion method for minimizing both deflection and tissue damage during insertion into multilayered tissues is beneficial. The optimum parameter of automatic acupuncture needle insertion will depend on the insertion velocity, tissue characteristics, and tissue structure. The penetration depth of the needle may be adjusted automatically in conjunction with sensors that determine a depth as the needle can penetrate without contacting blood vessels or nerves. The safety issue of an automanipulation device for acupuncture operation in rats is feasible among real-time physiological functions and laboratory data, and no significant adverse effect was noted such as crippling and molting in the whole process [57].

On the other hand, the visual feedback of the medical robot system is also an important factor for the accuracy of needle insertion. AcuBot consists of a small surgical module capable of needle orientation for manipulating a needle in the confined space based on aligning the needle with the laser marks [58]. Its structure provides flexibility in performing interventions at any location of the body and with different imaging modalities, which is also applicable for acupuncture. The robot moves automatically to position the tip of the needle, with the predetermined angle, at the skin entry point [59]. The cadaver study shows the feasibility of using this system in a basic joystick experiment. Robotic insertion of various ablation needles was accurate regardless of the type of needle or location in swine as the overall mean accuracy was 2.8 mm [17]. The average diameter of a single acupoint on the limbs and torso was 10 mm [60]. Such accuracy shows a promising future for more automatic devices on acupuncture [61, 62], which can contribute to the judgement of the needle position and provide efficient insertion strategy.

#### 2.2.3. Needle Manipulation and User Interaction

Manipulation features of an acupuncture robot can be designed based on the observation of common interventions performed by experienced acupuncturists.

Acupuncture needle manipulation involves sophisticated hand movements and represents a fundamental skill for acupuncturists, which also influence the curative effect. The automatic robot manipulation algorithm should be designed and developed by selecting characteristic manipulations from numerous acupuncturists and acupuncture academicians. An acupuncture device includes a needle-wakening method for driving the acupuncture needle to rotate alternately in forward and reverse directions when the interval of time has arrived [63]. Such devices can implement the basic manipulation of acupuncture, and by integration of simple movement, the complex manipulation can be replicated.

Robotic needle insertion has many advantages such as safety and better user experience. Needle rotation may be an effective method of reducing targeting errors as well as reducing the pain of insertion, which is favorable by changing a higher static friction between tissue and needle to a lower kinetic friction and then reducing insertion forces. Actuated needle drivers can incorporate other useful features such as rotation. Several experiments on animals have proven that insertion with vibration decreases the insertion force and torque, while insertion with bidirectional rotation decreases deflection and avoid wind up [64]. Needle rotation can improve targeting and may reduce errors by as much as 70% [65].

User interaction with an acupuncture robot can be mediated through the user's feedback. Separate mechanisms with independent control can be used to perform horizontal and vertical positioning, orientation, and instrument insertion such as visual feedback and force feedback. An acupuncture robot can improve the sophisticated movements required for acupuncture needle manipulation using sensor motor learning during acupuncture needle manipulation using visual feedback [66]. Pupillary reaction as a feedback of acupuncture varies depending on the different stimulation acupoints [67].The combined finite-element and kinematic analysis led to an improved performance according to repeated positioning accuracy and curative effect [68]. The section has a predetermined level of flexibility, enabling the robot system's limited movement [69]. The arm section has high rigidity, such that the pose of the end actuator relative to the patient is accurately maintained [70]. The mechanical parameters can be fitted with high accuracy, demonstrating the ability of the model to reproduce the mechanical coupling due to the presence of multiple directional reinforcements [71]. Furthermore, the robot can be controlled by motions of a user's head, shoulders, and ankles, enabling a host of assistive interactions with the curative actions [72]. Additionally, an emergency stop button should be available to stop the treatment immediately.

### 2.3. Force Feedback and Evaluation for Acupuncture Stimulation

In manual acupuncture stimulation, an aching sensation appeared to be the most predominant as Deqi sensation [73], followed by sharp pain and tingling sensations. Deqi is defined in relation to acupuncture needling as a sensory perception of varying character [74]. The complex pattern of sensations in the Deqi response involves a wide spectrum of nerve fibers. Deqi has been proven to be of great significance in the difference of the neurophysiological mechanism between acupuncture responders and nonresponders [75], which can be quantified through force feedback technology and so on.

#### 2.3.1. Force Feedback

Although the robot mainly relies on visual feedback from real-time images during needle insertion, force feedback can confer another safety benefit. Prudent acupuncture techniques are commonly practiced with the intent to avoid large neurovascular structures, thereby minimizing potential excessive bleeding and neural injury [76]. Besides, traditional Chinese acupuncturists rely on the force feedback by hand to make sure whether the treatment is effective too.

Innovative technological developments within the framework of biomedical research are used in the fields of complementary medicine to make it more accessible to younger acupuncturists by lowering the required manual skills threshold. In fact, many practitioners who want to become professional acupuncturists lack practical training in performing acupuncture for anxiety about deep needling, lack feedback from actual patients, etc. An acupuncture training system based on a force feedback device can help [77]. The ability to analyze feedback from patients is also essential for an acupuncture robot.

The force feedback mechanism for an acupuncture robot is different from that for other medical robots. The robotic arm is configured to operate on the patient in a variety of positions [78]. It is difficult to directly observe the state of the needle after inserted into the skin [40]. For most surgical robotics, needle insertion is usually performed under CT guidance by manual controller manipulation of the physician, while the future acupuncture robot needs to rely on itself. The force of the needle penetration should be restrained while the force feedback data can be gathered through the intelligent needle with a sensor operatively coupled to the adjustment mechanism. The sensor is adapted to determine a penetration depth, which is an amount the needle can penetrate the skin while avoiding blood vessels [79]. The method for controlling force and body scan in real time should control the vertical height of the acupuncture part [80]. An analytical force model can successfully estimate the force and the depth in experimental conditions [81].

Finite-element simulation analysis can verify the rationality of the deformation of the flexible driving unit [82]. A tactile sensing instrument, which uses a commercially available sensor to measure distributed pressure profiles along the contacting surface, has realized a 55% decrease in the maximum forces applied on tissue, a 50% decrease in task completion time, and a 40% increase in acupoint detection accuracy [83]. With the combination of models and sensors, automatic force feedback for an acupuncture robot will come true in the near future.

#### 2.3.2. Evaluation for Acupuncture Stimulation

The automatic force feedback mechanism is not the only way to detect the acupoint accurately; multiparameter and multimode are the trend of the modern acupoint sensing system. Available modules include an ultrasound sensor to capture ultrasound images, a pressure sensor to sense the pressure exerted by the robotic arm on the acupoint, a touch sensor to detect the hardness or firmness of the treatment spot, a temperature sensor to detect the temperature of the treatment area [84], a soft laser unit, and a skin-resistance-measuring device for localizing acupoints [85], which is under theoretical guidance from acupoint sensitization [86, 87].

Acupoint sensitization has variation in the mechanism and pattern. An organ's states are reflected in real time at its related acupoints, causing physical, real-time changes in the local tissue. Many separate details of the organ's function are reflected at acupoints [88]. The diagnostic points when being palpated and presenting sensitivity reactions may indicate imbalance in the related visceral organs [89]. Most acupoints are located near peripheral nerves, neuromuscular junctions, blood vessels, and ligaments, and this contributes to their low electrical resistance in the skin and conduction of energy. It has been found that the phase space of the time series corresponding to the electrical activity of neurons is embedding parameters, which implies that the neuronal signals are chaotic [90]. Needling signal is a spatiotemporal grouped sequence of input neuroinformation [91]. There has been a robot, which could monitor evolving changes of the body such as moles that may turn into melanoma or skin irritations that may indicate episodes of psoriasis [92]. Building the characterization mechanism of the acupoint diagnosis based on an acupuncture robot is helpful to promote the deepness of meridian theory.

### 2.4. Other Methods of Stimulation

Insertion of needles is not the only form of acupuncture treatment; numerous stimulation methods work for quantifying the effects of acupuncture. Acupuncture therapy includes magnetic needles, electronic beam, etc. The acupuncture robot may be featured with more kinds of stimulation, such as acupressure, electrical, and laser therapy [93, 94]. Further experiments should focus on exploring uniform criteria for selecting parameters for those advanced medical technologies [95]. Nowadays, electrical stimulation devices at specific acupoint areas [96] have evolved into advanced implants which can stimulate peripheral activity to relieve conditions (SPARCs). This program seeks to accelerate development of therapeutic devices that modulate electrical activity in nerves to improve organ function. Numerous inventions have been successfully put into use [97, 98]. Those devices can target the peripheral nervous system and perform neuromodulation noninvasively [99]. The electronic signals produced by the built-in chip contain the afferent signal of neuroinformation encoding currents, meeting the needs of bioinformation feedback therapy [100]. The principle of stimulation is similar to acupuncture. Laser stimulation is an optical, pain-free stimulation method while high-tech acupuncture research has been performed for more than 10 years as animal experiments have been performed in pigs, dogs, and sheep [101]. Innumerous clinical studies have shown that low-level lasers are safe and lack side effects [102, 103]. A laser watch for simultaneous laser blood irradiation and laser acupuncture at the wrist has been developed [104]. A trial in volunteers shows that yellow laser acupuncture is a new option for prevention and early intervention of lifestyle-related diseases [105], as a salutary attempt for the future of laser acupuncture robot.

Other than the noninvasive acupuncture therapy, an innovative design of the acupuncture device may enable the robot to perform the catgut-embedding procedure [106]. An acupuncture needle delivery system that facilitates the sanitary insertion and handling of the acupuncture needle in all orientations [107] would also work for an acupuncture robotic system for fully automatic acupuncture.

To sum up, numerous stimulation and measurement methods that work for quantifying the effects of acupuncture will identify potential candidates for the effects of acupuncture and provide valuable information toward understanding the possible mechanisms of the therapy.

### 2.5. Intelligentization of Acupuncture Prescription

The appropriate selection of acupoints is fundamental to obtain a therapeutic effect from clinical acupuncture. It is not easy to remember the precise locations of hundreds of acupoints and their corresponding therapeutic effects without extensive training. However, there is a possibility for intelligent acupuncture robot.

Artificial intelligence enables automatic extraction of relevant information, contributing to better decision making and intervention guidance for acupuncture [108]. Thousands of years' clinic practices in acupuncture have accumulated a considerable number of prescriptions that exhibit reliable in vivo efficacy and safety. The prescription of acupuncture includes the following parts: firstly, the robot needs to figure out what acupoints needed to be stimulated and how much stimulation would be enough. Secondly, the robot needs to decide on the manipulation method that ensures the curative effect of acupuncture. From the published works, we concluded that the most sophisticated part for acupuncture prescription is how to choose acupoints automatically according to existing medical classics. The selection of acupoints is a nonlinear process which is far more different from the way that Western medicine does. A single acupoint is not merely linked to one symptom or disease. A collection of acupoints may focus on the treatment of one symptom. Using the modified mutual information technique, a systematic framework for the acupoint combination network can reveal how the technique of acupoint combination is used in the treatment of specific diseases [109]. However, the problem as the existed symptom-acupoint relationship cannot be directly put into use.

Luckily, there are a few works [110] to figure out how to transform the existed symptom-acupoint relationship into practical acupuncture prescription. In our view, this progress might be the most difficult one to be digitized for acupuncture robot. The prescription of acupuncture contains the Chinese thought patterns and cannot easily be defined as a linear relationship. The digital representation of the symptom-acupoint complex network serves as a basis for decision making. One of the feasible ways to reveal the hidden orders behind the traditional Chinese medicine thinking method is to digitize the evidence-based clinical data of acupuncture. Several teams have independently developed their symptom-acupoint systems to reveal the nonlinear relationship among acupuncture prescriptions [111, 112]. It is urgent to utilize existing information technology to dynamically integrate these heterogeneous data from ancient classics and recent works to make medical information sharing efficient.

Systems biology, which attempts to understand the function by studying the interactions between components, will shed light on the automatic acupoint combination as well as the mystery of meridian. The meridian network is designed by various coupling acupoints, which could be perturbed by actuating stimulus. The nervous network functionally connects the distant parts of the system. The metabolic networks, the neural information exchange, and the long-range correlated structures of information exchange in the living organisms work on the WWW style [113]. The development of network-based systems biology has provided considerable support for the understanding of the holistic, complementary, and synergic essence of acupuncture [114]. Without further holistic and sophisticated analyses in the context of systems biology, the current attempts and the foreseeable developments appear to be insufficient to produce firm conclusions [115]. The analysis of acupuncture data with computational technologies provides biological evidence for the basic understanding of mechanisms, safety, and efficacy of acupuncture treatments. The accumulation of a massive amount of biomedical data makes knowledge-intensive computation technologies more important [116], which ensures a more scientific way of acupuncture prescription.

With effective co-operation with the abovementioned components, the complete design of medical acupuncture robot would become reality as will be discussed below. These parts provide a systematic understanding and pave the way for further experimental explorations on the intelligent acupuncture equipment.

## 3. Complete System Design of Acupuncture Robot

Besides teams from other part of the world, teams from China have focused on complete system design of acupuncture robot from the 1990s till now. According to available research, the team from Luzhou Medical College developed the first feasible acupuncture robot of its kind in 1993 [117]. A few teams have developed prototypes in the early 2000s [118, 119]. More research work in this field, thoroughly and synthesized, was carried on recently by teams from Shanghai Jiao Tong University [120], Tianjin University [121], and other research teams [122, 123], and a comparison of the most representative studies is shown in Table 1. A fully implemented design of acupuncture robot seems achievable.

The efforts of many researchers have led to significant advancements in the design, application, and theoretical foundation for acupuncture robot, as has been reviewed in previous sections of this paper. However, before the acupuncture robot becomes as practical as a real acupuncturist, a number of ongoing research challenges must be addressed. Inspired by the abovementioned analysis, we attempt to touch on the problems mentioned above through two emblematic cases [124].

**Figure 2:**
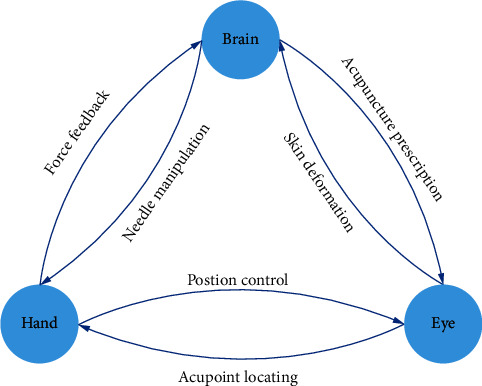
Hand-eye-brain coordination of an acupuncture robot.

Comprehensively speaking, the complete system design of an acupuncture robot should conclude a hand-eye-brain coordination framework. The hand represents the robot arm, the eye represents the visual sensor, and the brain represents the control center, which writes out a prescription as well as ensures safety. We need to recognize the interdependence of three key factors. The acupuncture robot can be completed only when the three coordination are well organized, as Figure 2 shows.

## 4. Summary

In summary, the acupuncture robot offers the potential to extend the benefits of acupuncture to applications that have not previously been approachable with acupuncturists. Most of the acupuncturists need much practice to perform acupuncture painlessly. The acupuncture robot can make it a more pleasant progress by pricking the needle at a high speed effortlessly. The unpleasant pain of acupuncture prevents many potential clients of such therapy. Furthermore, the robot can implement more kinds of manipulations than electronic acupuncture devices. The widely used electroacupuncture apparatus can only lift the needle up and down, without the necessary rotation that may increase the curative effect of acupuncture. While the acupuncture robot arm with multiple degrees of freedom may perform those manipulations by receiving electric signals from the acupuncturist, it is modeled on and can record curative data for every patient to set suitable treatment mode personally.

However, medical acupuncture robot has not proceeded to commercialization, but its clinical impact and innovation would be highlighted soon. Although, several teams have separately developed acupuncture robot or its related technologies and even went to the stage of clinical trial and then achieved success commercially. The acupuncture robot that can achieve a comprehensive automation is still unachievable. The reason lies on three aspects: (1) the force feedback technology for clinical use is not mature enough; (2) the automatic acupuncture prescription with evidence-based medicine remains elusive; and (3) lack of a compatible platform for the integration of related technologies. It will also provide a great platform which makes communication between upstream and downstream players of the acupuncture industry more pragmatic and effective by new products of acupuncture [125, 126]. Last but not least, soft and flexible robots for medical applications are needed to change their flexibility over a wide range to perform tasks adequately [127], so as to acupuncture robot. Those technologies include a spatially resolved soft sensor for biomechanics and robotic haptic capability which may lead to better interaction between patients and the robot [128]; also, the use of self-disclosure and forward lean by a healthcare robot can increase human engagement and attentional behaviors [129]. More intelligent components would be integrated in order to achieve integration of a plurality of functions required in medical acupuncture applications.

## Figures and Tables

**Table 1:** Comparison of related work.

University of inventors	Shanghai Jiao Tong University	National Cheng Kung University
Acupoint locating	A visual servoing task with correlation filter algorithm	Combine facial landmarks and image deformation to estimate acupoints
Path planning	A hierarchical control strategy of the image Jacobian matrix	Formulated as a hand-eye calibration problem
Insertion control	By judging the threshold value of evaluation criteria	Not mentioned
Feedback of stimulation	A device to transform the deformation of the skin into the deformation of the spring	A device based on collection of electroencephalogram signals to automatically detect a Deqi effect
Human-robot interaction	The magnitude of the force can be estimated through visual observation of skin deformation	The user interacts with a chatbot to describe her/his symptoms
